# A Case of Uterine Hemorrhage Following the Expulsion of the Placenta

**Published:** 1849-04

**Authors:** L. P. Yandell


Art. II.—*A Case of Uterine Hemorrhage following the expulsion of the Placenta.* By L. P. Yandell, M.D.

The following case is submitted to our readers, not because it possesses anything novel, but because it illustrates some practical points in obstetrics which cannot be too well understood.

Mrs. M., aged 32 years, the mother of two children, was brought to bed with her third at 10 o'clock on the night of the 27th of February. I was called to see her at 3 o'clock in the morning, five hours after the labor commenced, and on examination found everything favorable. The vertex was presenting, the os uteri was considerably dilated, soft and yielding, and but for the representation of the patient that her previous labors had been tedious, and the fact that her pains were then slight, I should have promised her a speedy delivery. The circumstances of the case favoring it, I had recourse repeatedly to my friend, Professor Miller's favorite expedient of orificial irritation; but, either for want of skill, or from not persevering long enough, I did not succeed in bringing on efficient uterine contractions. At 10 o'clock, twelve hours after the beginning of labor, I was not sure that the head had made any progress, or that the pains had increased. I was obliged to be absent for some hours, and at 11 o'clock my son, Dr. D. W. Yandell, took charge of the case. He informs me that, for more than two hours, the pains continued feeble and inefficient; the membranes were never distended, and, with the pressure of his finger nail that it appeared to him proper to employ, he was not able to rupture them. About half past 1 o'clock a violent, long-continued pain came on, followed in a few seconds by another which expelled the head. The membranes came before it and were ruptured as the head was about being born. I delivered the placenta in about twenty minutes after the birth of the child, the uterus feeling hard and well contracted.

We left our patient in apparently a safe and comfortable condition, and did not see her again until 6 o'clock in the evening, when I called and learned that, soon after we left her chamber, flooding came on and had been profuse. Her face was blanched and she was nearly pulseless, but the hemorrhage seemed to have ceased. I applied my hand over the abdomen and felt the womb contracted into a ball. The bandage was reapplied as tightly as was comfortable, and sixty drops of laudanum given. Her pulse rose, and for half an hour things appeared to be going on well, when symptoms of syncope returned. I resolved now to ascertain the cause of the hemorrhage, and accordingly introduced my hand into the vagina and pushed it gently forward into the cavity of the uterus, which I found filled with coagula. It was contracted pretty firmly upon its contents, and I found some difficulty in turning my hand about. The friction necessary to detach the coagula brought on efficient contractions, and on withdrawing my hand the coagula were forcibly expelled after it. There was no further hemorrhage, and the woman suffered less than was to have been expected from so serious a loss of blood. In fact, she had a good recovery.

The evils in this case originated in the first stage of the labor, which was unnecessarily tedious; the consequence was the too rapid progress of the second stage, and the hemorrhage which followed the separation of the placenta. Orificial irritation continued for a longer time and with more energy, might have accelerated the first stage; and it is certain that advantage would have been derived from rupturing the membranes at an earlier period. The expulsive efforts of the womb would thereby have been increased, and the fetus being born, the tonic contraction would have been more prompt and complete. The only remedy that would have availed in the state of things which existed when I saw the patient at six o'clock in the evening was the one resorted to—the introduction of the hand into the cavity of the uterus.

March 15, 1849.

